# Blocking Bacterial Naphthohydroquinone Oxidation and ADP-Ribosylation Improves Activity of Rifamycins against Mycobacterium abscessus

**DOI:** 10.1128/AAC.00978-21

**Published:** 2021-08-17

**Authors:** Uday S. Ganapathy, Tian Lan, Philipp Krastel, Marissa Lindman, Matthew D. Zimmerman, HsinPin Ho, Jansy P. Sarathy, Joanna C. Evans, Véronique Dartois, Courtney C. Aldrich, Thomas Dick

**Affiliations:** a Center for Discovery and Innovation, Hackensack Meridian Health, Nutley, New Jersey, USA; b Department of Medicinal Chemistry, University of Minnesota, Minneapolis, Minnesota, USA; c Natural Products Unit, Novartis Institutes for Biomedical Research, Novartis Pharma AG, Basel, Switzerland; d Department of Medical Sciences, Hackensack Meridian School of Medicine, Nutley, New Jersey, USA; e Department of Microbiology and Immunology, Georgetown University, Washington, DC, USA

**Keywords:** *Mycobacterium abscessus*, rifamycin, rifabutin, rifampicin, ADP-ribosylation, bacterial cell pharmacokinetics

## Abstract

Rifampicin is an effective drug for treating tuberculosis (TB) but is not used to treat Mycobacterium abscessus infections due to poor *in vitro* activity. While rifabutin, another rifamycin, has better anti-M. abscessus activity, its activity is far from the nanomolar potencies of rifamycins against Mycobacterium tuberculosis. Here, we asked (i) why is rifabutin more active against M. abscessus than rifampicin, and (ii) why is rifabutin’s anti-M. abscessus activity poorer than its anti-TB activity? Comparative analysis of naphthoquinone- versus naphthohydroquinone-containing rifamycins suggested that the improved activity of rifabutin over rifampicin is linked to its less readily oxidizable naphthoquinone core. Although rifabutin is resistant to bacterial oxidation, metabolite and genetic analyses showed that this rifamycin is metabolized by the ADP-ribosyltransferase Arr_Mab_ like rifampicin, preventing it from achieving the nanomolar activity that it displays against M. tuberculosis. Based on the identified dual mechanism of intrinsic rifamycin resistance, we hypothesized that rifamycins more potent than rifabutin should contain the molecule’s naphthoquinone core plus a modification that blocks ADP-ribosylation at its C-23. To test these predictions, we performed a blinded screen of a diverse collection of 189 rifamycins and identified two molecules more potent than rifabutin. As predicted, these compounds contained both a more oxidatively resistant naphthoquinone core and C-25 modifications that blocked ADP-ribosylation. Together, this work revealed dual bacterial metabolism as the mechanism of intrinsic resistance of M. abscessus to rifamycins and provides proof of concept for the repositioning of rifamycins for M. abscessus disease by developing derivatives that resist both bacterial oxidation and ADP-ribosylation.

## INTRODUCTION

Since its discovery in 1965, rifampicin has played a critical role in the treatment of tuberculosis (TB). The addition of rifampicin to the TB drug regimen helped shorten treatment time to 6 months and reduced the relapse rate (1). The efficacy of rifampicin can be attributed to several desirable antimycobacterial properties. An inhibitor of the beta-subunit of mycobacterial RNA polymerase (RpoB) (2–4), rifampicin has bactericidal activity against Mycobacterium tuberculosis both within macrophages (5) and in caseous lesions (6, 7). Rifampicin also retains activity against drug-tolerant, nonreplicating bacterial subpopulations (8). While these properties have made rifampicin indispensable in the treatment of TB, rifampicin’s utility in treating lung infections caused by nontuberculous mycobacteria (NTM) is less clear-cut. Although rifampicin is prescribed to treat lung infections by Mycobacterium kansasii and members of the Mycobacterium avium complex (MAC), it is not recommended for treatment of Mycobacterium abscessus lung disease due to observed poor *in vitro* activity (9–11). Whereas rifampicin resistance in M. tuberculosis is associated with the acquisition of *rpoB* mutations, clinical isolates of M. abscessus do not have *rpoB* mutations (12), suggesting that this mycobacterium has intrinsic resistance to rifampicin. In fact, M. abscessus is innately resistant to a broad range of antibiotics, including all first-line TB drugs (13, 14). For this reason, current M. abscessus treatment relies on macrolide-based combination regimens instead (15, 16). These regimens, however, are increasingly compromised by acquired and inducible macrolide resistance (17, 18) and have poor bactericidal activity (19). Unsurprisingly, current M. abscessus treatment does not provide a reliable cure (20). The absence of potent, bactericidal agents like rifampicin has made M. abscessus lung disease one of the most difficult bacterial infections to treat.

Rifabutin, an analog of rifampicin, was recently reported to be 5- to 10-fold more active against M. abscessus *in vitro* (21). In fact, rifabutin is active against all three subspecies of the M. abscessus complex (M. abscessus subsp. *abscessus*, M. abscessus subsp. *massiliense*, and M. abscessus subsp. *bolletii*) as well M. abscessus isolates that are resistant to clarithromycin (21, 22). As an RNA polymerase inhibitor, rifabutin synergizes with clarithromycin by inhibiting transcription of *whiB7* and *erm41*, effectively suppressing inducible macrolide resistance by M. abscessus (23–25). Rifabutin’s anti-M. abscessus activity is bactericidal and is retained against intramacrophage bacteria (21, 26–29). Crucially, rifabutin was recently shown to be just as active as clarithromycin against M. abscessus in a mouse infection model, whereas rifampicin had no *in vivo* activity (30). A recent clinical report has also described rifabutin-azithromycin combination therapy as effective in treating disseminated M. abscessus infections in immunodeficient adults (31). Taken together, these findings suggest rifabutin as a repurposing candidate for M. abscessus lung disease (32). However, a recent study found that the *in vitro* susceptibility of 200 M. abscessus complex clinical isolates to rifabutin was both variable and skewed toward higher MICs (clinical MIC_50_ and MIC_90_ values of 19 and 38 µM, respectively) (33), indicating that a more potent rifamycin will ultimately be needed for effective treatment (34).

While rifabutin is more active against M. abscessus than rifampicin, its potency still pales in comparison to its anti-TB activity. Rifabutin displays nanomolar potency against M. tuberculosis but only has an MIC of 1 to 4 µM against M. abscessus complex (8, 21). Stranger still, rifabutin and rifampicin are equally potent against M. tuberculosis (8). What accounts for the reduced and differential potencies of rifampicin and rifabutin against M. abscessus? A difference in on-target activity is unlikely given that the coding sequences of RpoB are largely conserved between M. abscessus and M. tuberculosis, including the four regions associated with rifamycin resistance (35). Instead, rifabutin and rifampicin may be subject to differential bacterial cell pharmacokinetics in M. abscessus: differences in drug uptake, efflux, and/or metabolism by M. abscessus could alter intrabacterial rifamycin levels and, thus, whole-cell activity (34). As M. abscessus possesses a diverse array of antibiotic-inactivating enzymes (13), differential bacterial metabolism is a likely suspect. In fact, M. abscessus encodes a homolog of ADP-ribosyltransferase (*MAB_0591*, *arr_Mab_*), which can inactivate rifamycins by conjugating ADP-ribose to the C-23 position of the scaffold (36–38). Deletion of *arr_Mab_* improved the potency of rifamycins (35, 39). Thus, ADP-ribosylation by Arr_Mab_ contributes to poor M. abscessus activity of this drug class.

In this study, we sought to answer the following two questions: (i) why is rifabutin more active against M. abscessus than rifampicin, and (ii) why is rifabutin’s anti-M. abscessus activity poorer than its anti-TB activity? We found that rifabutin’s improved potency against M. abscessus is linked to the increased oxidative stability of the naphthoquinone core versus the more reactive naphthohydroquinone moiety found in rifampicin and rifapentine. We also showed that rifabutin undergoes ADP-ribosylation in M. abscessus, preventing this drug from achieving the nanomolar potency that it has against M. tuberculosis. With this knowledge in hand, we screened a large collection of rifamycins and found derivatives that were more potent against M. abscessus than rifabutin. Structure-activity relationship analysis attributed the improved potency of these compounds to the following two features: a more stable naphthoquinone core and modifications that block ADP-ribosylation. Our findings define the roles of two intrinsic rifamycin resistance mechanisms in M. abscessus and provide proof of concept for the repositioning of rifamycins for M. abscessus lung disease by blocking enzymatic oxidation and ADP-ribosylation.

## RESULTS

### Improved anti-M. abscessus activity of rifabutin versus rifampicin is linked to its more chemically stable naphthoquinone core.

**FIG 1:**
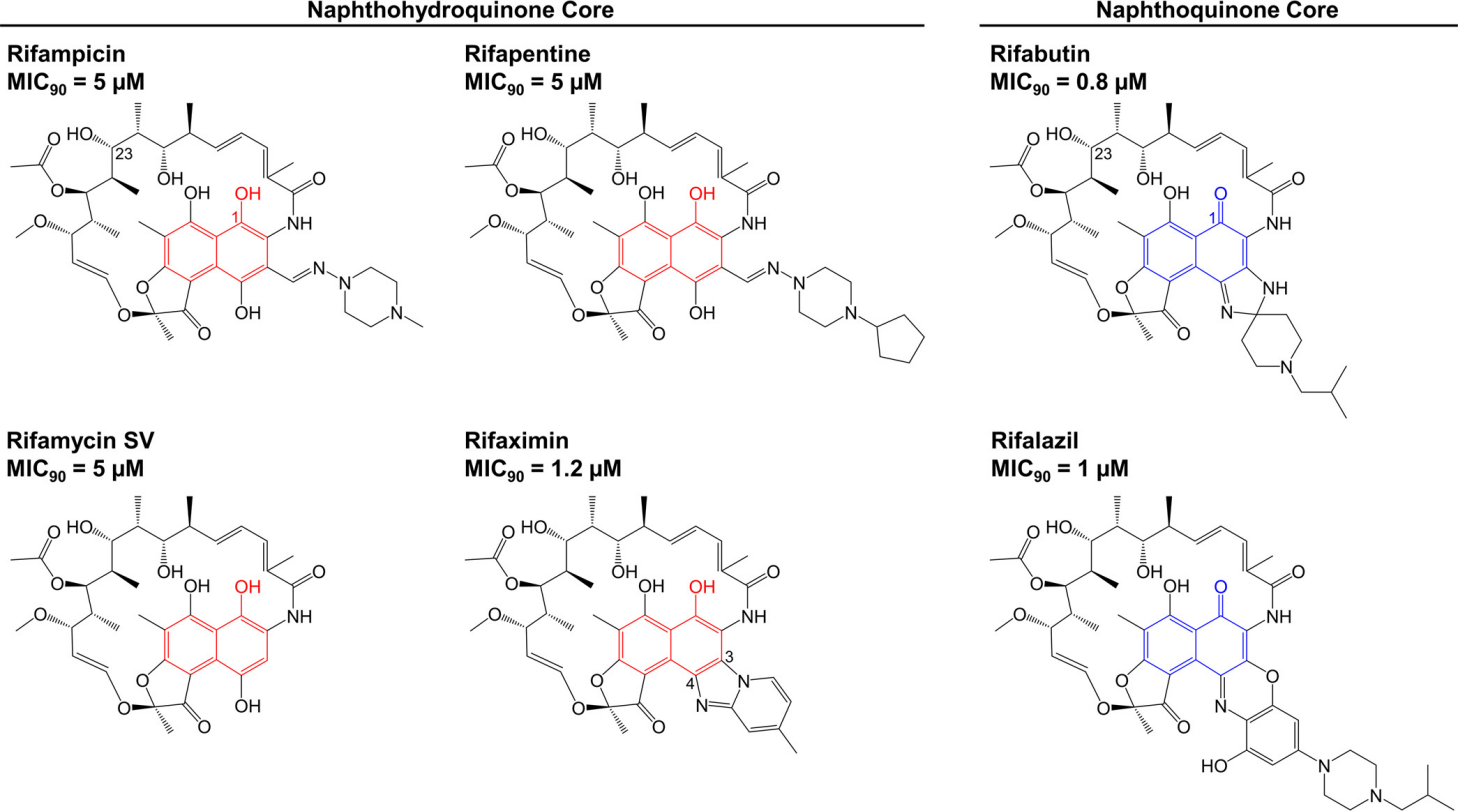
Rifamycin potency in M. abscessus correlates with the presence of a naphthoquinone core. Structures of six rifamycins and their MICs against M. abscessus Bamboo. The structures of rifabutin and rifalazil have a naphthoquinone core with a C-1 carbonyl group (blue). The structures of rifampicin, rifapentine, rifamycin SV, and rifaximin have a naphthohydroquinone core with a C-1 hydroxyl group (red). The C-1 and C-23 positions of the rifamycin scaffold are indicated in the rifampicin and rifabutin structures. The C-3 and C-4 positions are indicated in the structure of rifaximin. MIC values are the mean of two independent experiments.

The poorly active rifampicin contains an electron-rich and more readily oxidizable naphthohydroquinone core (Fig. 1) (21). Interestingly, the rifamycin analog rifapentine also has a naphthohydroquinone core and, like rifampicin, has poor anti-M. abscessus activity (21). In contrast, the more potent rifabutin features an electron-deficient and less-oxidizable naphthoquinone core (Fig. 1). Genome analyses showed that M. abscessus—but not M. tuberculosis—harbors at least five potential homologs of the Streptomyces venezuelae rifamycin monooxygenase (see Table S1 in the supplemental material) (39, 40). This *S. venezuelae* enzyme was shown to oxidize and inactivate naphthohydroquinone-containing rifamycins, whereas naphthoquinone analogs are not substrates (40). Thus, we hypothesized that rifabutin’s naphthoquinone core may play a role in its increased potency against M. abscessus compared to that of rifampicin/rifapentine by preventing enzymatic redox inactivation of the molecule. To test this hypothesis, we determined the potency of additional rifamycins containing either a naphthoquinone or a naphthohydroquinone core (Fig. 1) (41). The naphthoquinone-containing rifalazil showed good activity that was comparable to that of rifabutin (Fig. 1). In contrast, the naphthohydroquinone-containing rifamycin SV showed poorer potency like rifampicin and rifapentine (Fig. 1). Thus, the presence of an electron-deficient and less easily oxidized naphthoquinone core appears to be associated with greater rifamycin potency. If the differential potency of naphthoquinone versus naphthohydroquinone rifamycins is indeed due to differential metabolism, the intrabacterial level of the latter should be lower. To measure intrabacterial levels, we treated M. abscessus cultures with the rifamycins and determined drug concentrations by liquid chromatography-tandem mass spectrometry (LC-MS/MS) (see Fig. S1 in the supplemental material). The more potent, naphthoquinone-containing rifabutin and rifalazil achieved high levels inside the bacterium (Fig. S1). Consistent with being metabolized, rifamycins containing a naphthohydroquinone core (rifampicin, rifapentine, and rifamycin SV) showed 10- to 20-fold lower intrabacterial concentrations (Fig. S1).

In an attempt to determine the enzyme(s) responsible for the inactivation of naphthohydroquinone rifamycins, we deleted the top-scoring candidate M. abscessus rifamycin monooxygenase MAB_0857 (see Table S1 and Fig. S2 in the supplemental material). However, the deletion did not affect susceptibility of M. abscessus to rifamycins (see Table S2 in the supplemental material). During the course of this work, Schäfle et al. deleted MAB_0857 as well as two additional candidate M. abscessus rifamycin monooxygenases (MAB_3483 and MAB_1496c, including a double knockout of MAB_0857 and MAB_3483) (Table S1) without observing any effects on the susceptibility to rifamycins (39). Together, these results suggest redundancy in the putative rifamycin monooxygenase activity of M. abscessus, i.e., several enzymes may have the capacity to oxidize naphthohydroquinone-containing rifamycins (39). Alternatively, other (unknown) enzymes are responsible for the oxidation and inactivation of naphthohydroquinone rifamycins in M. abscessus. Thus, the enzymatic basis of the apparent oxidative inactivation of naphthohydroquinone-containing rifamycins remains to be determined.

Taken together, our comparative analyses of naphthohydroquinone- versus naphthoquinone-containing rifamycins suggest that the improved anti-M. abscessus activity of rifabutin over rifampicin is linked to its less easily oxidized naphthoquinone core.

### Rifabutin’s anti-M. abscessus potency is limited by ADP-ribosylation.

**FIG 2:**
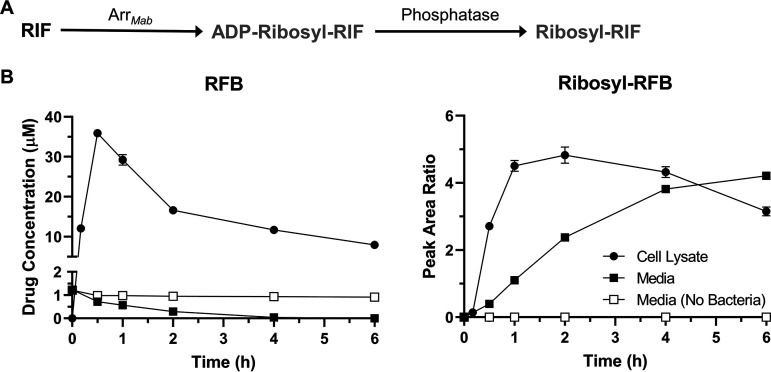
Rifabutin undergoes ADP-ribosylation in M. abscessus. (A) M. abscessus expresses ADP-ribosyltransferase Arr*_Mab_*, which can conjugate ADP-ribose to rifamycins, resulting in drug inactivation. Subsequent phosphatase activity results in ribose-conjugated rifamycins. (B) LC-MS/MS detection of rifabutin (RFB) and ribosyl-rifabutin (Ribosyl-RFB) in M. abscessus Bamboo cultures treated with 1 µM rifabutin for 6 h. Compound levels in the cell lysate (filled circles), media (filled squares), and in media lacking bacteria (empty squares) were measured. For rifabutin, compound levels in the cell lysate were expressed as intrabacterial concentration. For ribosyl-rifabutin, compound levels from the cell lysate and media were expressed as peak area ratios relative to the LC-MS/MS internal standard.

M. abscessus expresses the ADP-ribosyltransferase Arr_Mab_ (35). The MICs of several rifamycins decreased in an M. abscessus Arr_Mab_ deletion mutant, demonstrating that M. abscessus ADP-ribosyltransferase activity can limit the potency of rifamycins (35), including rifabutin (39). To confirm that rifabutin is subject to ADP-ribosylation, we carried out a metabolite analysis. ADP-ribosyltransferases like Arr_Mab_ convert rifamycins into ADP-ribose conjugates (37, 38) (Fig. 2A). These ADP-ribose conjugates, however, can be further metabolized by phosphatases, resulting in ribose-conjugated rifamycins (42–44) (Fig. 2A; see also Fig. S3 in the supplemental material). If rifabutin is ADP-ribosylated by Arr_Mab_, we would expect M. abscessus to produce ribose-conjugated rifabutin after exposure to this rifamycin. To test this, M. abscessus cultures were incubated with rifabutin and cell lysates, and media samples were analyzed by LC-MS/MS. Initially, after drug addition, the intrabacterial concentration of rifabutin increased rapidly, peaked after 30 min, and then declined (Fig. 2B). The levels of rifabutin in the culture media decreased steadily over time (Fig. 2B). This depletion of rifabutin from the media was consistent with drug uptake by M. abscessus, as rifabutin levels were stable in media lacking bacteria (Fig. 2B). Starting at 10 min after drug addition, we detected a compound with a mass-to-charge ratio corresponding to ribose-conjugated rifabutin (ribosyl-rifabutin, observed *m/z *= 979.494) in both the M. abscessus cell lysates and the culture media (Fig. 2B). The cell lysate levels of ribosyl-rifabutin increased over the first 2 h post-drug addition before reaching a plateau. The levels of ribosyl-rifabutin in the culture media increased unabated over the time course of the experiment. Production of ribosyl-rifabutin was bacteria dependent, as we did not detect ribosyl-rifabutin in media lacking M. abscessus (Fig. 2B). Based on these results, rifabutin is ADP-ribosylated by M. abscessus within minutes of being added to the culture. Bacterial phosphatases convert ADP-ribosyl-rifabutin to ribosyl-rifabutin, which can then be effluxed by M. abscessus into the culture media. We also detected ribose-conjugates for the other rifamycins that we analyzed for potency (Fig. 1; see also Table S3 in the supplemental material), suggesting that all tested rifamycins, whether they contain a naphthoquinone or a naphthohydroquinone core, are subject to ADP-ribosylation.

**FIG 3:**
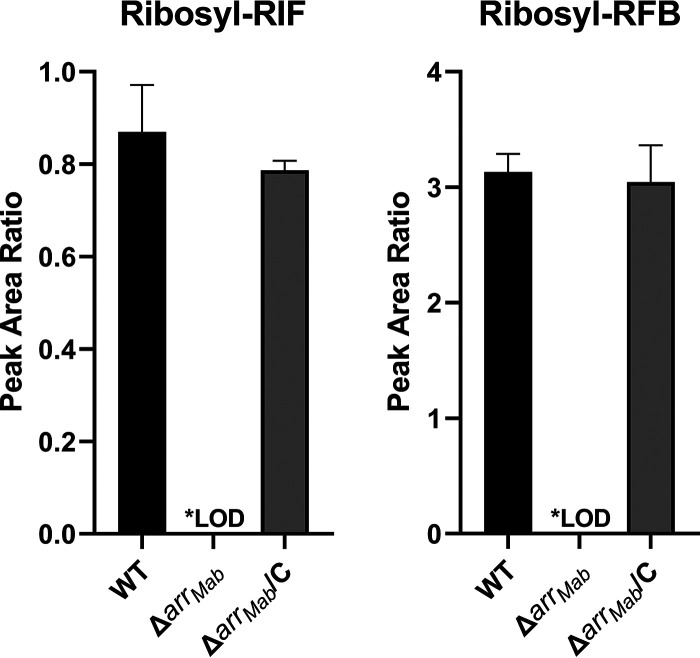
*arr_Mab_* is required for the production of rifamycin ribose-conjugates in M. abscessus. LC-MS/MS detection of ribosyl-rifampicin and ribosyl-rifabutin in cell lysates from M. abscessus ATCC 19977, Δ*arr_Mab_*, and Δ*arr_Mab_*/C strain cultures treated with 10 µM rifampicin or rifabutin for 30 min. The mean and standard deviation of triplicate measurements are plotted. LOD, below the limit of detection.

Since rifabutin undergoes ADP-ribosylation in M. abscessus (Fig. 2B), we asked whether the ADP-ribosyltransferase Arr_Mab_ (35) is responsible for this activity. To test this, we generated the M. abscessus Δ*arr_Mab_* strain, where *arr_Mab_* was deleted and replaced with an apramycin resistance cassette (see Fig. S4 in the supplemental material). We did not detect ribosyl-rifampicin or ribosyl-rifabutin in drug-treated M. abscessus Δ*arr_Mab_* strain cultures, demonstrating that ADP-ribosylation of rifamycins was abolished in this strain (Fig. 3). Detection of ribose-conjugated rifamycins was restored in the M. abscessus Δ*arr_Mab_*/C strain, a complementation strain of the Δ*arr_Mab_* strain that expresses *arr_Mab_* under the constitutive *hsp60* promoter (Fig. 3). Thus, *arr_Mab_* is required for ADP-ribosylation of rifabutin in M. abscessus.

To determine the effect of ADP-ribosylation on rifabutin’s anti-M. abscessus potency, we determined the MIC of rifabutin against the M. abscessus Δ*arr_Mab_* strain. Compared to its activity against wild-type (WT) M. abscessus, rifabutin was 63 times more potent against the Δ*arr_Mab_* strain with an MIC of 0.030 µM (Table 1) (39). Similarly, rifampicin was 43-fold more potent against the Δ*arr_Mab_* strain (MIC = 0.22 µM) than the parental strain (Table 1). Potency shifts were also observed for the other four rifamycins previously tested, while the potency of the macrolide clarithromycin was unchanged (Table 1). The shifts in the potency for rifabutin and other rifamycins were reversed by restoring expression of *arr_Mab_* in the Δ*arr_Mab_* strain background (Table 1, Δ*arr_Mab_*/C strain MICs). Thus, rifabutin’s potency against M. abscessus is reduced significantly by the bacterium’s ADP-ribosyltransferase activity. These genetic results are consistent with a recent study analyzing the effect of deletion of *arr*_Mab_ on the activity of rifabutin (39). It is noteworthy that rifabutin remained more potent against the M. abscessus Δ*arr_Mab_* strain than rifampicin (Table 1). This difference in potency is consistent with rifampicin’s susceptibility to enzymatic oxidation, which would still occur in the M. abscessus Δ*arr_Mab_* strain. Indeed, other naphthohydroquinone-containing rifamycins (rifapentine, rifamycin SV, and rifaximin) were also less potent against the Δ*arr_Mab_* strain than rifabutin (Table 1). Notably, only the naphthoquinone-containing rifamycins (rifabutin and rifalazil) achieved nanomolar potency against the Δ*arr_Mab_* strain (Table 1), resembling the potency of rifamycins against M. tuberculosis (8). Therefore, anti-TB-like rifamycin activity can be achieved against M. abscessus if both enzymatic oxidation and ADP-ribosylation are blocked.

**TABLE 1:** ADP-ribosylation limits the potency of rifabutin against M. abscessus

Drug^*b*^	MIC_90_ (µM)^*a*^		
	WT^*c*^	Δ*arr_Mab_* strain	Δ*arr_Mab_*/C strain
Rifamycin, hydroquinone naphthalene core			
RIF	9.5	0.22	6.8
RFP	10	0.28	4.8
RSV	7.5	1.1	4.6
RFX	2.2	0.39	1
Rifamycin, quinone naphthalene core			
RFB	1.9	0.030	0.68
RFL	1.6	0.052	0.57
Macrolide			
CLR	2.1	1.7	1.8

a MIC values are the mean of two independent experiments.

b RIF, rifampicin; RFP, rifapentine; RSV, rifamycin SV; RFX, rifaximin; RFB, rifabutin; RFL, rifalazil; CLR, clarithromycin.

c M. abscessus ATCC 19977.

Our comparative analyses of activity and intrabacterial accumulation of naphthoquinone versus naphthohydroquinone rifamycins showed that naphthohydroquinone-containing rifamycins are poorly active and show low-level intrabacterial concentration, consistent with their inactivation by bacterial redox activities (Fig. 1 and Table 1; see also Fig. S1). Interestingly, we did observe an exception to this trend in the case of rifaximin. Although rifaximin’s structure contains a naphthohydroquinone (Fig. 1) and should therefore be inactivated by enzymatic oxidation (40), this rifamycin analog was surprisingly potent (Table 1) and attained a high intrabacterial level (Fig. S1). Unexpectedly, rifaximin showed only a modest (6-fold) increase in potency when tested for activity against the M. abscessus Δ*arr_Mab_* strain (Table 1). This result suggests that rifaximin undergoes less ADP-ribosylation than the other rifamycins, thus explaining the comparably high potency despite containing an oxidizable naphthohydroquinone core. In the absence of ADP-ribosyltransferase activity, rifaximin should still be susceptible to enzyme-mediated oxidation like other naphthohydroquinone-containing scaffolds. Indeed, rifaximin’s MIC against the M. abscessus Δ*arr_Mab_* strain was comparable to those of the other naphthohydroquinone-containing rifamycins and 7- to 10-fold higher than the MICs of naphthoquinone-containing rifamycins (Table 1). Thus, rifaximin appears to achieve better potency against M. abscessus by evading ADP-ribosylation but is still subject to enzymatic oxidation. Since the structure of rifaximin lacks any modifications near the site of ADP-ribosylation at C-23 (Fig. 1), the analog’s unique C-3/C-4 side group (Fig. 1) may be responsible for reducing ADP-ribosylation.

### Rifamycins more potent than rifabutin contain rifabutin’s naphthoquinone core plus a modification that blocks ADP-ribosylation.

Our analyses suggest that M. abscessus metabolizes and thus inactivates naphthohydroquinone-containing rifamycins by the following two mechanisms: oxidation and ADP-ribosylation. To test this dual-metabolism model, we carried out a blinded screen of a diverse collection of 189 rifamycin analogs and predicted that, if analogs more potent than rifabutin can be identified, they would belong to the less oxidizable, naphthoquinone-containing rifamycins and would carry modifications near the ADP-ribosylation site at C-23 to block this enzymatic inactivation (35, 44, 45). Past efforts to optimize rifamycins for mycobacteria harboring *arr* have focused on blocking ADP-ribosylation at the C-23 hydroxyl by introducing modifications at the neighboring C-25 acetate group, which can be altered without loss of on-target activity (46). C-25 carbamate-linked rifamycin derivatives had enhanced activity against the nonpathogenic mycobacterial model organism Mycobacterium smegmatis that was also *arr*-independent, demonstrating successful blocking of ADP ribosylation in that mycobacterial species (45). However, this finding appears not to have translated effectively to M. abscessus, as the same C-25 carbamate rifamycins that blocked ADP-ribosylation in M. smegmatis did not block this activity in M. abscessus (35, 44). Thus, blocking rifamycin ADP-ribosylation by mycobacteria appears to be species specific. Based on past works in M. smegmatis and M. abscessus (35, 44, 45), we speculated that more potent rifamycins identified in a screen against M. abscessus would carry novel, noncarbamate modifications at C-25.

**FIG 4:**
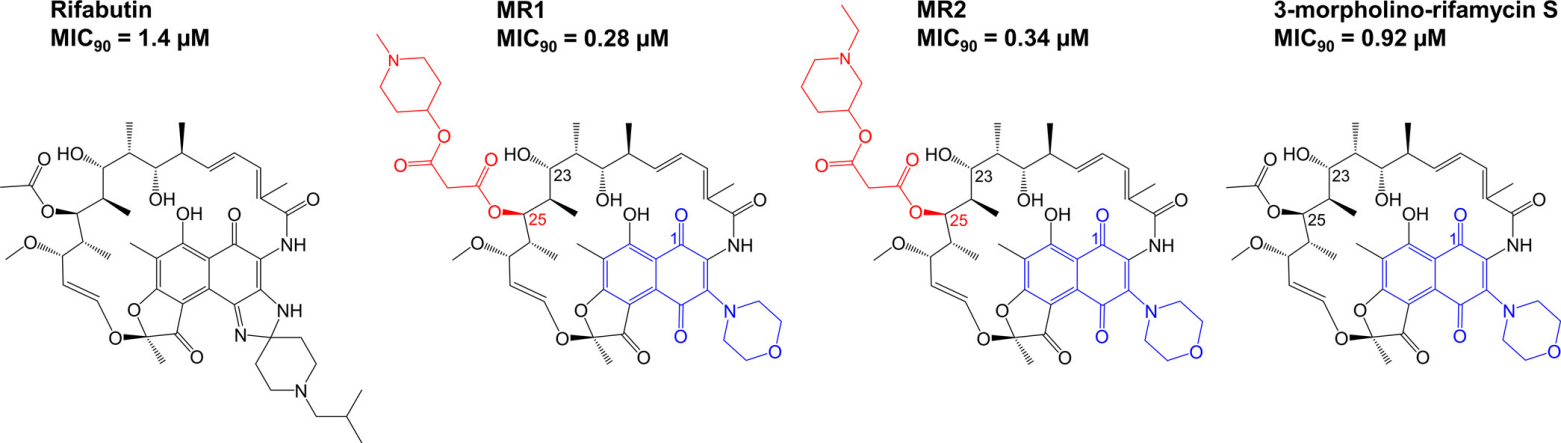
A screen identifies two rifamycins with greater potency against M. abscessus than rifabutin. A blinded, single-point screen of 189 rifamycins identified MR1 and MR2, which both have better potency against M. abscessus Bamboo than rifabutin. These compounds have the same oxidatively resistant naphthoquinone core (blue) and different modifications to the rifamycin scaffold at C-25 (red). 3-Morpholino-rifamycin S, the parent compound of MR1 and MR2, retains the same less-readily oxidizable naphthoquinone core but has no modifications at C-25. The C-1, C-23, and C-25 positions of the rifamycin scaffold are indicated in the structures of MR1, MR2, and 3-morpholino-rifamycin S. MIC values are the means of two independent experiments.

The blinded screen of 189 rifamycins against M. abscessus identified two hits, MR1 and MR2, that were more active than rifabutin (Fig. 4, Table 2). Both MR1 and MR2 were inactive against an M. abscessus rifabutin-resistant mutant strain carrying a missense mutation in RpoB (H447Y) corresponding to the extensively characterized H526Y mutation associated with rifampicin resistance in M. tuberculosis (35, 47), demonstrating that the activity of these compounds remained on-target (Table 2). Unblinding of the structures of the two hits revealed that MR1 and MR2 had features that are consistent with the proposed intrinsic rifamycin resistance mechanisms of M. abscessus (Fig. 4). Both compounds had a naphthoquinone core that would resist enzymatic oxidation (40) and carried novel malonate ester modifications at C-25 that may prevent ADP-ribosylation (37, 38).

**TABLE 2:** The anti-M. abscessus potencies of two C-25 malonate rifamycins are Arr_Mab_-independent and on-target

Drug^*b*^	MIC_90_ (µM)^*a*^			
	WT^*c*^	Δ*arr_Mab_* strain	Δ*arr_Mab_*/C strain	RFB-R1^*d*^
Rifamycin, hydroquinone naphthalene core				
RIF	9.1	0.18	8.3	>100
Rifamycin, quinone naphthalene core				
RFB	1.7	0.028	0.83	>100
MR1	0.25	0.11	0.27	>100
MR2	0.49	0.14	0.25	>100
3mo-RfS	1.2	0.060	0.64	92
Macrolide				
CLR	1.4	1.2	1.5	2.2

a MIC values are the mean of two independent experiments.

b RIF, rifampicin; RFB, rifabutin; 3 mo-RfS, 3-morpholino-rifamycin S; CLR, clarithromycin.

c M. abscessus ATCC 19977.

d RFB-R1 has a RpoB H447Y mutation that was previously reported in M. tuberculosis (35, 47).

To determine the contributions of the naphthoquinone core and C-25 malonate ester modifications to the anti-M. abscessus activities of MR1 and MR2, we characterized their parent compound, 3-morpholino-rifamycin S (Fig. 4). 3-Morpholino-rifamycin S has the same naphthoquinone core as MR1 and MR2 (Fig. 4) but lacks their modifications at C-25. Based on these structural features, 3-morpholino-rifamycin S should not be subject to enzymatic oxidation but be susceptible to ADP-ribosylation. Thus, the molecule should show increased potency compared to that of rifampicin (as it cannot be oxidized) but reduced potency compared to that of MR1 and MR2 (as it is subject to ADP-ribosylation); that is, its potency should be rifabutin-like. Indeed, 3-morpholino-rifamycin S had rifabutin-like potency against M. abscessus as predicted (Fig. 4 and Table 2).

To demonstrate directly that the C-25 modifications in MR1 and MR2 block ADP-ribosylation (45), we measured the potencies of MR1, MR2, and 3-morpholino-rifamycin S against the M. abscessus Δ*arr_Mab_* strain. The MIC of 3-morpholino-rifamycin S decreased 20-fold against the Δ*arr_Mab_* strain relative to that of the wild-type strain (Table 2). Therefore, the potency of this compound against M. abscessus is severely limited by ADP-ribosylation. In contrast, both MR1 and MR2 showed no significant shift in potency (Table 2), demonstrating that their C-25 modifications improved potency by blocking ADP-ribosylation. Taken together, both the naphthoquinone core and C-25 modifications of MR1 and MR2 were required for their enhanced anti-M. abscessus activity, with each feature accounting for one of the bacterium’s two intrinsic rifamycin resistance mechanisms.

## DISCUSSION

Given the excellent track record of rifamycins as TB drugs, the poor activity of this compound class against another mycobacterial pathogen, M. abscessus, is puzzling. Not only do rifamycins lack their nanomolar anti-TB potency against M. abscessus, but some rifamycins (e.g., rifabutin) are more active against this bacterium than others (e.g., rifampicin). In this study, we sought to understand the basis for these discrepancies in the anti-M. abscessus activity of rifamycins.

Comparative analysis of whole-cell activity and intrabacterial concentrations of naphthoquinone- versus naphthohydroquinone-containing rifamycins (Fig. 1; see also Fig. S1 in the supplemental material) suggested that the improved activity of rifabutin compared to that of rifampicin is linked to its less readily oxidizable naphthoquinone core. Rifamycins containing an oxidizable naphthohydroquinone core showed lower antibacterial activity and lower intrabacterial concentrations, consistent with the hypothesis that they are intracellularly redox-inactivated. The enzymatic activities responsible for the redox inactivation of hydroquinone-containing rifamycins remain to be determined. M. abscessus harbors at least five (see Table S1 in the supplemental material) (39) putative homologs of rifamycin monooxygenase, an enzyme activity recently identified in *S. venezuelae* (40). These enzymes present plausible candidates to exert this function (39, 40). However, an M. abscessus strain carrying a deletion of two of these candidates did not show an alteration in susceptibility to hydroquinone-containing rifamycins (Table S1) (39). This could indicate that several of the candidate rifamycin monooxygenases are capable of inactivating rifamycins or that other enzymes are responsible for this activity. Although rifabutin is resistant to bacterial oxidation, metabolite (Fig. 2) and genetic (Table 1) (39) analyses showed that this drug, like other rifamycins (see Table S3 in the supplemental material), is metabolized in M. abscessus by the ADP-ribosyltransferase Arr_Mab_, preventing it from achieving the nanomolar activity that it displays against M. tuberculosis. Together, these results explain why rifabutin is more active than rifampicin against M. abscessus as well as why the activity of rifabutin against M. abscessus is far weaker than its activity against M. tuberculosis. Crucially, rifabutin had an MIC of 30 nM against the Δ*arr_Mab_* strain (Table 1), demonstrating that this compound would achieve the nanomolar potency that it has against M. tuberculosis (8) if it were not susceptible to ADP-ribosylation. It is interesting to note that naphthohydroquinone-containing rifamycins were at least 10 times less potent against the M. abscessus Δ*arr_Mab_* strain than against M. tuberculosis (Table 1) (8), further highlighting that a rifamycin must avoid both enzymatic oxidation and ADP-ribosylation to achieve anti-TB-like activity against M. abscessus.

To test the dual metabolism model of intrinsic rifamycin resistance in M. abscessus, we carried out a blinded screen of a library of 189 diverse rifamycins. The model predicts that hits more potent than rifabutin should contain a less readily oxidizable naphthoquinone and modifications that block ADP-ribosylation. We identified two hits more potent than rifabutin (Fig. 4; Table 2). Unblinding revealed that both hits indeed contained a naphthoquinone core (Fig. 4). Furthermore, both otherwise structurally identical hits contained different malonate ester modifications at C-25. Previous work in M. smegmatis had shown that carbamate modifications at C-25 block ADP-ribosylation in this mycobacterial species, without elimination of on-target activity (45). However, these C-25 carbamate analogs did not prevent ADP-ribosylation in M. abscessus, suggesting species specificity (35). To determine whether the C-25 malonate modifications are indeed responsible for blocking ADP-ribosylation in M. abscessus, we determined the activity of the two hits and their parent compound lacking a C-25 modification in the M. abscessus Δ*arr_Mab_* strain, which lacks ADP-ribosyltransferase activity. Whereas the activity of the two C-25-modified hits did not change in the Δ*arr_Mab_* strain, the parent compound’s activity increased strongly in this loss-of-function mutant (Table 2). This showed that the malonate ester modifications are indeed responsible for blocking ADP-ribosylation. The parent compound showed rifabutin-like activity against wild-type M. abscessus strains, making it superior to rifampicin and supporting the role of its naphthoquinone core in preventing oxidation. Thus, our structure-activity relationship analysis suggests that the improved potency profiles of the two hits required the following two structural features: a naphthoquinone core to provide rifabutin-like potency by blocking enzyme-mediated oxidation and (malonate ester) modification of the C-25 hydroxyl to further enhance activity by blocking ADP-ribosylation.

The identification of MR1 and MR2 in our screen provides proof of concept for the development of a rifamycin for M. abscessus lung disease by blocking intrabacterial metabolism (34). Starting from these hit compounds, SAR determination will be needed to develop a lead compound with not only greater anti-M. abscessus potency but the same desirable properties that rifampicin has as an anti-TB drug. Therefore, future studies will evaluate selected lead rifamycin compounds for improved bactericidal activity against M. abscessus (19) as well as potency against the bacterium when it resides in macrophages (28) or forms biofilms (48). Critically, any lead compound will need to retain desirable pharmacokinetic properties, have low cytotoxicity, and ultimately demonstrate improved *in vivo* efficacy in an animal infection model before becoming a candidate for preclinical development (30).

In conclusion, we have linked the poor and variable potency of rifamycins against M. abscessus to two metabolic activities, namely, enzymatic oxidation and ADP-ribosylation, both of which result in inactivation of this compound class. We further demonstrated that modifications to the rifamycin scaffold that block these activities can improve anti-M. abscessus potency, supporting the repositioning of this drug class. As such, our work describes how a potent TB drug class (the rifamycins) has been rendered ineffective against M. abscessus, an emerging mycobacterial pathogen, by differences in bacterial cell pharmacokinetics (34). Just as pharmacokinetics determine antibiotic efficacy at the host level, bacterial cell pharmacokinetics dictate drug potency at the level of the bacterium. Beyond drug uptake and efflux mechanisms, the role of bacterial metabolism in limiting cellular drug concentration and ultimately potency is gaining recognition both in M. abscessus (13) and other bacterial species (49, 50). As such, we expect the optimization of drug bacterial cell pharmacokinetics to not only become a critical component of M. abscessus drug discovery but drive antibacterial drug discovery in the age of antibiotic resistance.

## MATERIALS AND METHODS

### Bacterial strains, culture media, and compounds.

M. abscessus Bamboo was isolated from the sputum of a patient with amyotrophic lateral sclerosis and bronchiectasis and was provided by Wei Chang Huang, Taichung Veterans General Hospital, Taichung, Taiwan. M. abscessus Bamboo whole-genome sequencing showed that the strain belongs to M. abscessus subsp. *abscessus* and harbors an inactive clarithromycin-sensitive *erm*(41) C28 sequevar (41, 51). Mycobacterium abscessus subsp. *abscessus* ATCC 19977, harboring the inducible clarithromycin resistance-conferring *erm*(41) T28 sequevar (36), was purchased from the American Type Culture Collection (ATCC).

For general bacteria culturing and MIC experiments, Middlebrook 7H9 broth (BD Difco) supplemented with 0.5% albumin, 0.2% glucose, 0.085% sodium chloride, 0.0003% catalase, 0.2% glycerol, and 0.05% Tween 80 was used. Solid cultures were grown on Middlebrook 7H10 agar (BD Difco) supplemented with 0.5% albumin, 0.2% glucose, 0.085% sodium chloride, 0.5% glycerol, 0.0003% catalase, and 0.006% oleic acid.

Clarithromycin, rifamycin SV, and rifaximin were purchased from Sigma-Aldrich. Rifampicin was purchased from GoldBio. Rifapentine and rifabutin were purchased from Acros Organics. Rifalazil was purchased from BioDuro. The library of rifamycins, including MR1, MR2, and 3-morpholino rifamycins S, was provided by Novartis. All drugs were prepared as 10 mM stocks in 100% dimethyl sulfoxide (DMSO).

### MIC assay in 96-well plate format.

MIC determination was carried out in 96-well plate format as previously described (21, 52). The 96-well plates were initially set up with 100 µl of 7H9 per well. For each compound, a 10-point 2-fold dilution series starting at twice the desired highest concentration was dispensed onto the 96-well plates using a Tecan D300e digital dispenser, with the DMSO concentration normalized to 2%. M. abscessus culture grown to mid-log phase (optical density at 600 [OD_600_] = 0.4 to 0.6) was diluted to an OD_600_ of 0.1 (1 × 10^7^ CFU/ml). One hundred microliters of the resulting bacterial suspension was dispensed onto the 96-well plates containing compounds to give a final volume of 200 µl per well with an initial OD_600_ of 0.05 (5 × 10^6^ CFU/ml) and a final DMSO concentration of 1%. Final compound concentration ranges were typically 50 to 0.098 µM or 6.25 to 0.012 µM but were adjusted to 100 to 0.195 µM for testing of the rifamycin-resistant strain RFB-R1. Untreated control wells are included on each plate that contains a bacterial suspension and 1% DMSO. Plates were sealed with parafilm, stored in boxes with wet paper towels, and incubated at 37°C with shaking (110 rpm). Plates were incubated for 3 days. To determine growth, OD_600_ was measured using a Tecan Infinite M200 plate reader on day 0 and day 3. Two biological replicates were performed. Clarithromycin was included in each experiment as a positive control.

For each well on the 96-well plate, bacterial growth was calculated by subtracting the day 0 OD_600_ value from the day 3 OD_600_ value. For each compound series, the bacterial growth values for the untreated control wells were averaged to give the average drug-free bacterial growth. For compound-containing wells, percentage growth was calculated by dividing their growth values by the average drug-free bacterial growth for the compound series and multiplying by 100. For each compound series, we plotted percentage growth versus compound concentration. By visual inspection of the dose-response curve, we determined the MIC of a compound as the compound concentrations that would result in 90% growth inhibition.

### Identification of candidate M. abscessus ROX homologs.

The amino acid sequence of rifamycin monooxygenase ROX from Streptomyces venezuelae ATCC 10712 (UniProt accession number F2R776) was used as a query in an NCBI BLASTP search against nonredundant protein sequences from the Mycobacterium abscessus ATCC 19977 genome. Search results were filtered based on an E-value cutoff of 1E−20 and ranked by alignment max score. The hits were used as queries in additional BLASTP searches against the Mycobacterium tuberculosis H37Rv ATCC 27294 genome to determine the presence or absence of M. tuberculosis homologs.

### Measurement of rifamycins in bacteria and culture media.

The method described was adapted from a previous study (53). M. abscessus culture grown to mid-log phase (OD_600_ = 0.4 to 0.6) was centrifuged at 2,400 × *g* for 10 min, and the cell pellet was resuspended in fresh 7H9 medium to achieve a concentrated cell suspension with an OD_600_ of 4.0 (OD 4.0 cell suspension, 1 × 10^9^ to 5 × 10^9^ CFU/ml). The OD 4.0 cell suspension was aliquoted into 15-ml Falcon tubes (5 to 7 ml per tube). Either 1 mM or 10 mM drug stocks prepared in 100% DMSO were added to each tube to achieve a final drug concentration of 1 µM or 10 µM, respectively, and a final DMSO concentration of 0.1%. The tubes were immediately vortexed and incubated on an Eppendorf ThermoMixer C at 37°C and 500 rpm. At each time point, a 1-ml sample of each drug-treated cell suspension was transferred to 2-ml Eppendorf tubes and centrifuged (21,000 × *g*, 5 min, 4°C) to pellet the cells. The top 500 µl of the supernatant was collected as a medium sample, which was stored at −20°C until LC-MS/MS analysis was performed. The cell pellet was washed twice with 1 ml cold phosphate-buffered saline (PBS) to remove extracellular drug. The cell pellet was then extracted by resuspension in a cold 1 ml acetonitrile/methanol 50/50 protein precipitation solvent containing internal standards at 10 ng/ml (internal standards are described in “LC-MS/MS analytical methods”) followed by mechanical cell lysis in screw-cap tubes containing 200 µl of 0.1-mm zirconia beads. Cell lysis was achieved by bead beating four times at 4,500 rpm for 30 s with samples kept on ice for 5 min between beatings. After bead beating, the beads and cell debris were removed by centrifugation, and the supernatant was collected as the final extracted cell lysate, which was stored at −20°C until LC-MS/MS analysis was performed. For the bacteria-free control, 5 to 7 ml of 7H9 medium in 15-ml Falcon tubes was treated with drug (final drug concentration of 1 µM) and incubated alongside the tubes containing the drug-treated OD 4.0 cell suspension. Samples were removed at each time point and stored at −20°C until LC-MS/MS analysis was performed. CFU measurement of the OD 4.0 cell suspension was performed at the 0 h time point. Specifically, serial 10-fold dilutions were prepared in phosphate-buffered saline (Thermo Fisher; 10010023) containing 0.05% Tween 80 (PBS/Tween 80) and plated on 7H10 agar.

For matrix-matching of LC-MS/MS standards to the cellular extracts, drug-free extracted cell lysate was generated by doing the same sample processing method (i.e., centrifugation, two PBS washes, extraction, and bead-beating) with 1-ml samples of the OD 4.0 cell suspension that had not been treated with drugs. The supernatant recovered after bead-beating and centrifugation was pooled and used to matrix-match LC-MS/MS standards for quantification of drugs in the cellular extracts (see “LC-MS/MS analytical methods”).

To determine the intrabacterial concentration of rifamycins, we quantified the nanomolar drug concentrations in the extracted cell lysates (*C*_lys_) by LC-MS/MS using standard curves generated for each rifamycin (see “LC-MS/MS analytical methods”). Based on a 1-ml sample volume of the drug-treated cell suspensions, we calculated the nanomolar intrabacterial drug concentration (*C*_i_) using the following formula: *C*_i_ = (*C*_lys_) ÷ (1,000 liters^−1^ × CFU × *V*_cell_), where CFU is the number of cells in 1 ml of the OD 0.4 cell suspension and *V*_cell_ is the aqueous volume of one mycobacterial cell (5 × 10^−16^ liters) (53).

Due to the lack of commercially available standards for ribose-conjugated rifamycins, the levels of ribosyl-rifampicin and ribosyl-rifabutin were expressed as peak area ratios relative to the LC-MS/MS internal standard (see “LC-MS/MS analytical methods”).

### LC-MS/MS analytical methods.

Neat 10 mM DMSO stocks of rifamycins were serial diluted in 50/50 acetonitrile and water (MeCN/H_2_O) to create neat spiking solutions. Extracted cell lysate standards and quality controls (QC) were created by adding 10 µl of neat spiking solutions to 100 µl of drug-free extracted cell lysates (preparation of the extracted cellular lysates is described in “Measurement of rifamycins in bacteria and culture media”). One hundred microliters of rifamycin-treated extracted cell lysates were spiked with 10 µl of MeCN/H_2_O. Samples were transferred to a 96-well plate for high-pressure liquid chromatography mass spectrometry (HPLC-MS) analysis. Culture medium standards were created by adding 10 µl of neat spiking solutions to 90 µl 7H9 medium. Culture media extraction was performed by adding 20 µl of control, standard, QC, or study medium to 200 µl of acetonitrile/methanol 50/50 protein precipitation solvent containing internal standards (IS) at 10 ng/ml. The labeled internal standards used were rifampicin-d8, rifapentine-d8, and rifabutin-d7, which were purchased from Toronto Research Chemical. Verapamil was used as the internal standard for rifaximin and rifalazil because labeled internal standards were not available. Labetalol was used for rifamycin SV. Verapamil and labetalol were purchased from Sigma-Aldrich. Extracts were vortexed for 5 min and centrifuged at 4,000 rpm for 5 min. One hundred microliters of extract was transferred to a 96-well plate for HPLC-MS analysis.

Liquid chromatography mass spectrometry (LC-MS) analysis was performed on a Q Exactive high-resolution mass spectrometer (QE-HRMS) at 70,000 mass resolution using an Ultimate 3000 ultrahigh-performance liquid chromatography (UHPLC) system for chromatographic separation. Full scan total ion chromatograms (TIC) of cell lysate and media extracts were acquired for quantitation of the rifamycins and for detection of the subsequent metabolites. Positive electrospray ionization mode was used for detection of all rifamycins except rifamycin SV, which was detected in negative mode. All analytes were detected within 5 ppm mass accuracy. Chromatography was performed on an Agilent SB-C_8_ column (2.1 × 30 mm; particle size, 3.5 µm) using a reverse-phase gradient. Milli-Q deionized water with 0.1% formic acid was used for the aqueous mobile phase and 0.1% formic acid in acetonitrile for the organic mobile phase. Data processing was performed using Thermo Xcalibur version 4.027.10.

Rifamycin SV LC-MS/MS analysis quantitation was performed on a Sciex Applied Biosystems Qtrap 6500+ triple-quadrupole mass spectrometer using negative-mode electrospray ionization. The MS system was coupled to a Shimadzu Nexera X2 UHPLC system. Rifamycin SV demonstrated low sensitivity on the Q-Exactive, and MS/MS analysis was required for improved sensitivity. Multiple-reaction monitoring of precursor/product transitions in electrospray positive-ionization mode was used to quantify the analytes. The following multiple reaction monitoring (MRM) transitions were used for rifamycin SV (697.00/273.00) and labetalol (327.20/176.00). Data processing was performed using Analyst software (version 1.6.2; Applied Biosystems Sciex).

### Generation and validation of *MAB_0857* and *MAB_0591* deletion mutants.

All vectors were generated by traditional cloning as described in Tables S4 and S5 in the supplemental material. To produce the *MAB_0857* and *arr_Mab_* allelic exchange substrates with an apramycin resistance cassette, we first generated pYUB854-apmR, a version of pYUB854 (54) in which the hygromycin resistance cassette was replaced with an apramycin resistance cassette. This required synthesis of pmK-T-apmR-Res1 (Invitrogen, Thermo Fisher Scientific), which contains *apmR* and a downstream Res1 site flanked by restriction sites as follows: SphI-*apmR*-NdeI-Res1-NcoI. The SphI-*apmR*-NdeI-Res1-NcoI fragment from pmK-T-*apmR*-Res1 was then used to replace the SphI-*hygR*-Res1-NcoI fragment excised from pYUB854 by restriction digestion/ligation. For genetic complementation, we generated pMV306hsp-*zeoR*, a version of pMV306hsp (55) in which the kanamycin resistance cassette was replaced with a zeocin resistance cassette under the control of the EM7 promoter. Apramycin was purchased from Alfa Aesar and used at 50 µg/ml. Zeocin was purchased from Thermo Fisher Scientific and used at 25 µg/ml.

The Δ*MAB_0857* and Δ*arr_Mab_* strains were generated by a recombineering strategy as described previously (56). For both *MAB_0857* and *arr_Mab_* (*MAB_0591*), ∼500-bp fragments corresponding to the regions upstream and downstream of these genes were amplified by PCR and cloned into pYUB854-*apmR* to flank the apramycin resistance cassette, generating pYUB854-*apmR*-MAB_0857KO and pYUB854-*apmR*-*arr_Mab_*KO (Tables S4 and S5). AflII/SpeI restriction digestion of the pYUB854-*apmR*-MAB_0857KO and pYUB854-*apmR*-*arr_Mab_*KO produced the *MAB_0857* and *arr_Mab_* allelic exchange substrates, which consisted of the apramycin cassette flanked by the upstream and downstream regions of either *MAB_0857* or *arr_Mab_*. M. abscessus ATCC 19977 was transformed with the recombineering vector pJV53-*zeoR* (gift from Anil Singh, North Eastern Regional Institute of Science and Technology, Nirjuli, India) to generate the appropriate recombineering strain. Mid-log phase (OD_600_ = 0.4 to 0.6) M. abscessus ATCC 19977(pJV53-*zeoR*) culture was induced by adding acetamide (final concentration = 0.2%) and incubating for 3 h at 37°C. After 3 h of incubation, the culture was used to prepare competent cells, which were then transformed with 100 ng of either the *MAB_0857* or *arr_Mab_* allelic exchange substrates and plated on 7H10 agar containing apramycin. Mutant genotypes were confirmed by PCR analysis (see Fig. S2 and S4 and Table S5 in the supplemental material). For complementation of the Δ*arr_Mab_* strain, we generated pMV306-*zeoR*-*arr_Mab_*, a vector that expresses *arr_Mab_* (*MAB_0591*) driven by the constitutive *hsp60* promoter and integrates into the attL5 site of the M. abscessus genome.

### Single-point screen of rifamycin library.

The single-point screen was carried as previously described (21, 52). Briefly, an M. abscessus Bamboo culture was grown to mid-log phase (OD_600_ = 0.4 to 0.6). The culture was diluted and dispensed onto 96-well plates containing compounds to give a final volume of 200 µl per well with 2 µM compound (final DMSO concentration of 1%) and an initial OD_600_ of 0.05 (5 × 10^6^ CFU/ml). Rifabutin was included on each plate as a positive control. Plates were sealed with a breathable membrane, stored in boxes with wet paper towels, and incubated at 37°C with shaking (110 rpm). Plates were incubated for 3 days. To determine growth, OD_600_ was measured using a Tecan Infinite M200 plate reader on day 0 and day 3. Percentage growth was calculated relative to the untreated control wells on the plate that contain bacterial culture and 1% DMSO. Two biological replicates of the screen were performed. Compounds with an average growth inhibition of 80% or higher were identified as hits. Hits were confirmed by determining their MIC against M. abscessus as described above.

### Selection of rifamycin-resistant M. abscessus mutant with mutation in *rpoB*.

Spontaneous resistant mutants were selected as described previously (57). Exponentially growing M. abscessus ATCC 19977 culture (10^8^ to 10^9^ CFU) was plated on 7H10 agar containing 32 µM rifabutin (32× MIC). The plates were incubated for 6 days at 37°C. Apparent resistant colonies were purified and confirmed by restreaking on agar containing the same concentration of drug. Genomic DNA was extracted as described previously using the phenol-chloroform method (58). Sanger sequencing of the *rpoB* (*MAB_3869c*) genomic region was performed by Genewiz (Genewiz, Inc., South Plainfield, NJ, USA) using 14 primers (Table S5). RFB-R1 carried an *rpoB* c1339t nucleotide mutation, which corresponded to an H447Y missense mutation that was previously reported in M. tuberculosis (35, 47).
