# Supplementary material for: Blocking Bacterial Naphthohydroquinone Oxidation and ADP-Ribosylation Improves Activity of Rifamycins against Mycobacterium abscessus
Source: Antimicrob Agents Chemother. 2021 Aug 17;65(9):e00978-21. doi: 10.1128/AAC.00978-21 (PMC8370238; doi:10.1128/AAC.00978-21)
Supplement: Supplemental file 1 — Supplemental material. Download AAC.00978-21-s0001.pdf, PDF file, 0.5 MB [file aac.00978-21-s0001.pdf]

Supplementary Information

Blocking bacterial naphthohydroquinone oxidation and ADP-ribosylation increases activity of rifamycins against *Mycobacterium abscessus*

Uday S. Ganapathy,[a] Tian Lan,[b] Philipp Krastel,[c] Marissa Lindman,[a*] Matthew D. Zimmerman,[a] HsinPin Ho,[a$] Jansy P. Sarathy,[a] Joanna C. Evans,[a] Veronique Dartois,[a,d] Courtney C. Aldrich,[b#] Thomas Dick[a,d,e#]

[a]Center for Discovery and Innovation, Hackensack Meridian Health, Nutley, New Jersey, USA

[b]Department of Medicinal Chemistry, University of Minnesota, Minneapolis, Minnesota, USA

[c]Natural Products Unit, Novartis Institutes for Biomedical Research, Novartis Pharma AG, Switzerland

[d]Department of Medical Sciences, Hackensack Meridian School of Medicine, Nutley, New Jersey, USA

[e]Department of Microbiology and Immunology, Georgetown University, Washington, DC, USA

#Address correspondence to Thomas Dick, thomas.dick@hmh-cdi.org; Courtney C. Aldrich, aldri015@umn.edu.

[*]Present address: Department of Cell Biology and Neuroscience, Rutgers University, Piscataway, NJ, USA

[$]Present address: Nonclinical Disposition and Bioanalysis, Bristol Myers Squibb, Princeton, New Jersey, USA

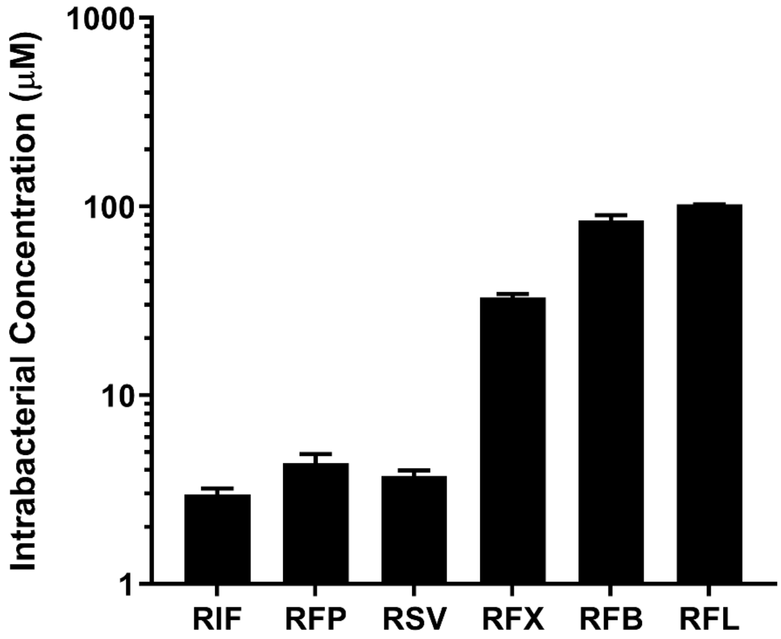

Figure S1. Rifamycins with naphthoquinone cores reach higher intrabacterial levels in *M. abscessus*. Intrabacterial levels of six rifamycins measured from *M. abscessus* Bamboo cultures that were treated with 10 µM of each rifamycin for 30 minutes, harvested for cell lysates and analyzed by LC/MS-MS. The mean and standard deviation of triplicate measurements are plotted. The structures of rifabutin (RFB) and rifalazil (RFL) have a naphthoquinone core whereas the structures of rifampicin (RIF), rifapentine (RFP), rifamycin SV (RSV), and rifaximin (RFX) have a naphthohydroquinone core.

A

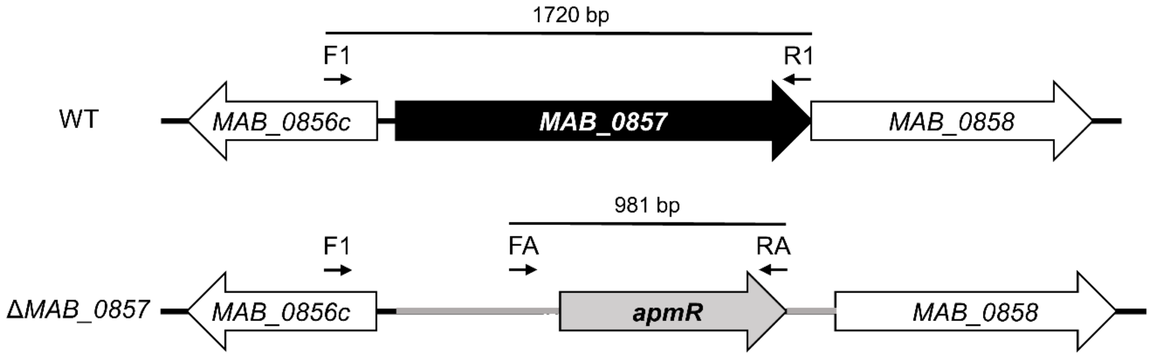

B

Primer Pair	WT	Δ*MAB_0857*
F1 + R1	1720 bp	No product
FA + RA	No product	981 bp

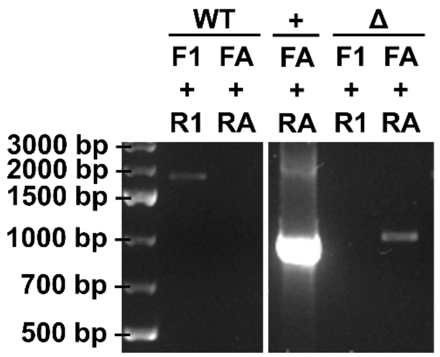

Figure S2. Generation and validation of *M. abscessus MAB_0857* deletion mutant.

(A) Strategy for deleting *MAB_0857* from *M. abscessus* ATCC 19977 (WT) by recombineering and validating Δ*MAB_0857* candidates by PCR analysis. **(B)** PCR analysis of WT and Δ*MAB_0857* (Δ) where genomic DNA was amplified with different primer pairs. For primer pair FA + RA, pYUB854-*apmR* plasmid DNA was used as a positive control (+) for amplification of *apmR*.

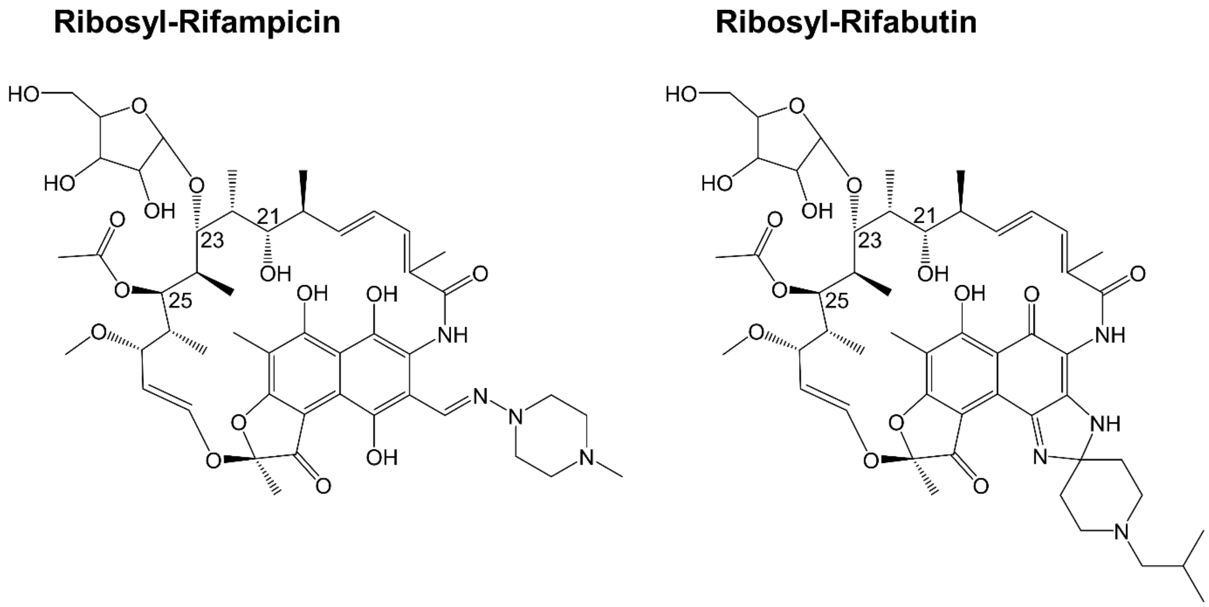

Figure S3. Structures of ribose-conjugated rifampicin and rifabutin. After rifamycins are ADP-ribosylated by Arr_{Mab} at the C23 hydroxyl, phosphatase activity results in ribose-conjugated rifamycins. Structures of 23-[O-(α-D-ribofuranosyl)] rifampicin (ribosyl-rifampicin) and 23-[O-(α-D-ribofuranosyl)] rifabutin (ribosyl-rifabutin) are shown. The C21, C23 and C25 positions of the rifamycin scaffolds are indicated.

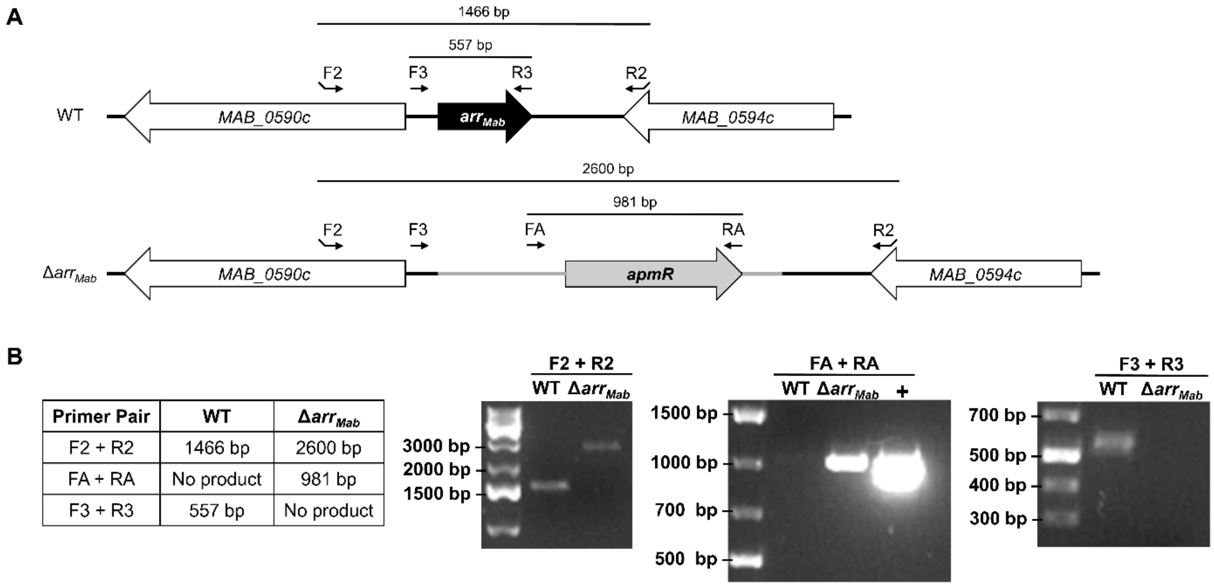

Figure S4. Generation and validation of *M. abscessus* $\textit{arr}_{\textit{Mab}}$ deletion mutant.

(A) Strategy for deleting $\textit{arr}_{\textit{Mab}}$ (*MAB_0591*) from WT *M. abscessus* ATCC 19977 by recombineering and validating $\Delta\textit{arr}_{\textit{Mab}}$ candidates by PCR analysis. **(B)** PCR analysis of WT *M. abscessus* ATCC 19977 and $\Delta\textit{arr}_{\textit{Mab}}$ where genomic DNA was amplified with different primer pairs. For primer pair FA + RA, pYUB854-*apmR* plasmid DNA was used as a positive control (+) for amplification of *apmR*.

Table S1. Results of protein BLAST of rifamycin monooxygenase ROX from *Streptomyces venzuelae* ATCC 10712 against proteins encoded by the *M. abscessus* ATCC 19977 genome[a]

Name	Annotation	Max Score	Query Coverage (%)	E-value	Identity (%)	*Mtb* homolog[b]
MAB_0857	Putative monooxygenase	373	98	3.00E-126	47.36	None
MAB_3483	Putative oxygenase	341	98	1.00E-113	42.19	None
MAB_1496c (TetX)	Flavin-dependent monooxygenase	191	94	5.00E-56	33.47	None
MAB_4357c	Putative monooxygenase	115	74	2.00E-28	31.52	None
MAB_2573c (MhpA)	3-(3-hydroxyphenyl)propionate hydroxylase	98.6	87	7.00E-23	26.41	None

[a]Results were filtered based on an E-value cutoff of 1E-20 and ranked by alignment max score.

[b]Presence or absence of a homologous protein in *M. tuberculosis* H37Rv is indicated.

Table S2. Potency of rifamycins against *M. abscessus* Δ*Mab_0857*

	MIC_{90} (μM)[a]	
**Drug[b]**	**WT[c]**	**Δ*MAB_0857***
RIF	9.5	14
RFP	10	11
RSV	7.5	10
RFX	2.2	2.6
RFB	1.9	1.7
RFL	1.6	1.3
CLR	2.1	2.0

[a]MIC values are the mean of two independent experiments.

[b]RIF, rifampicin; RFP, rifapentine; RSV, rifamycin SV; RFX, rifaximin; RFB, rifabutin; RFL, rifalazil; CLR, clarithromycin.

[c]*M. abscessus* ATCC 19977

Table S3. Rifamycin ribose conjugates detected in *M. abscessus* Bamboo whole cell lysates by LC-MS/MS[a]

			Exact mass (m/z)		
**Rifamycin[b]**	**Molecular Formula**	**ESI Ion Mode**	**Calculated**	**Observed**	**PPM**
**Naphthohydroquinone Core**					
Ribosyl-RIF	$C_{48}H_{67}N_4O_{16}$	Positive	955.4547	955.4572	2.6
Ribosyl-RFP	$C_{52}H_{72}N_4O_{16}$	Positive	1009.5016	1009.5035	1.9
Ribosyl-RSV	$C_{42}H_{55}NO_{16}$	Negative	826.3292	826.3370	9.4
Ribosyl-RFX	$C_{48}H_{60}N_3O_{15}$	Positive	918.4019	918.4048	3.2
**Naphthoquinone Core**					
Ribosyl-RFB	$C_{51}H_{71}N_4O_{15}$	Positive	979.491	979.494	3.1
Ribosyl-RFL	$C_{56}H_{72}N_4O_{17}$	Positive	1073.4965	1073.4967	0.2

[a]Lysates generated after 30-minute exposure of bacteria culture to 10 µM of drug.

[b]RIF, rifampicin; RFP, rifapentine; RSV, rifamycin SV; RFX, rifaximin; RFB, rifabutin; RFL, rifalazil.

Table S4. Vectors and cloning strategies used in this study.

Vector	AbxR[a]	Methodology	Vector	Restriction Sites	Template	PCR Primers[b]
pYUB854-*apmR*	Apm	Ligation	pYUB854	SphI/NcoI	pmK-T-*apmR*-Res1	
pYUB854-*apmR*-*arr*$_{Mab}$KO	Apm	1) PCR1: arr$_{Mab}$U	N/A		*M. abscessus* ATCC 19977 genomic DNA	Mab0591UFwd, Mab0591URev
		2) PCR2: arr$_{Mab}$D	N/A		*M. abscessus* ATCC 19977 genomic DNA	Mab0591DFwd, Mab0591DRev
		3) Ligation1: pYUB854-*apmR*-*arr*$_{Mab}$U	pYUB854-apmR	AflII/XbaI	PCR1	
		4) Ligation2: pYUB854-*apmR*-*arr*$_{Mab}$KO	Ligation1	HindIII/SpeI	PCR2	
pYUB854-*apmR*-MAB_0857KO	Apm	1) PCR1: MAB_0857U	N/A		*M. abscessus* ATCC 19977 genomic DNA	Mab0857UFwd, Mab0857URev
		2) PCR2: MAB_0857D	N/A		*M. abscessus* ATCC 19977 genomic DNA	Mab0857DFwd, Mab0857DRev
		3) Ligation1: pYUB854-*apmR*-MAB_0857U	pYUB854-apmR	AflII/XbaI	PCR1	
		4) Ligation2: pYUB854-*apmR*-MAB_0857KO	Ligation1	HindIII/SpeI	PCR2	
pMV306hsp-*zeoR*	Zeo	Subclone via PCR/ligation	pMV306hsp	NheI/SpeI	pGMCgZ-TSC28S38-P1-L5L[c]	EM7-zeoRFwd, EM7-zeoRRev
pMV306hsp-*zeoR*-MAB_0591	Zeo	1) PCR1: MAB_0591	N/A		*M. abscessus* ATCC 19977 genomic DNA	Mab0591pMV306Fwd, Mab0591pMV306Rev
		2) Ligation	pMV306hsp-zeoR	HindIII/SalI	PCR1	

[a]Apm, apramycin; Zeo, zeocin.

[b]Primer sequences are listed in Table S5.

[c]Gift from Carolina Trujillo (Weill Cornell Medical College, New York, USA). Contains zeocin resistance gene *ble* under control of the EM7 promoter.

Table S5. List of primers used in this study

Primer Name	Primer Sequence (5' to 3')[a]	Use in study
Mab0857UFwd	ACGTCTTAAGCTGCTCAATCGTGAAACCGATTGTG	pYUB854-*apmR*-MAB_0857KO
Mab0857URev	CTAGTCTAGAAACACTCCTTAACATCGTTATCGGCA	pYUB854-*apmR*-MAB_0857KO
Mab0857DFwd	CTCGAAGCTTTGTGGCCCAGGTGGCCGAGA	pYUB854-*apmR*-MAB_0857KO
Mab0857DRev	CTAGACTAGTAAGTACCGTCCAGGCTCACCCG	pYUB854-*apmR*-MAB_0857KO
Mab0591UFwd	ACGTCTTAAGTCACGGGTGGGCAGGAGGTA	pYUB854-*apmR*-*arr*$_{Mab}$KO
Mab0591URev	CTAGTCTAGAACCGGCTCCCATCAGTACAAATCGG	pYUB854-*apmR*-*arr*$_{Mab}$KO
Mab0591DFwd	CTCGAAGCTTCAGACGACCGGGGGCTATGC	pYUB854-*apmR*-*arr*$_{Mab}$KO
Mab0591DRev	CTAGACTAGTTGTACTTCAAGCGTGCTCACGGCAG	pYUB854-*apmR*-*arr*$_{Mab}$KO
EM7-zeoRFwd	ACGTGCTAGCTGTTGACAATTAATCATCGGCA	pMV306hsp-*zeoR*
EM7-zeoRRev	GATCACTAGTCTAGCAGTCCTGCTCCTCG	pMV306hsp-*zeoR*
Mab0591pMV306Fwd	ACGTAAGCTTATGACGATGCCCAACTTTTTCGAG	pMV306hsp-*ZeoR*-*arr*$_{Mab}$
Mab0591pMV306Rev	GATGGTCGACTCAGTCATAGATGACCGCGTTTCC	pMV306hsp-*ZeoR*-*arr*$_{Mab}$
FA	GCAGCTCACGGTAACTGATG	Genotyping PCR of Δ*MAB_0857* and Δ*arr*$_{Mab}$
RA	GCCAATCGACTGGCGAGC	Genotyping PCR of Δ*MAB_0857* and Δ*arr*$_{Mab}$
F1	GCCTCTTGAACGATCACCTC	Genotyping PCR of Δ*MAB_0857*
R1	CTACGCCCGAGAAGGTGATCC	Genotyping PCR of Δ*MAB_0857*
F2	ACGTCTTAAGTCACGGGTGGGCAGGAGGTA	Genotyping PCR of Δ*arr*$_{Mab}$
R2	CTAGACTAGTTGTACTTCAAGCGTGCTCACGGCAG	Genotyping PCR of Δ*arr*$_{Mab}$
F3	ACCGAGCGACTCTTCCAGTC	Genotyping PCR of Δ*arr*$_{Mab}$
R3	TCAGTCATAGATGACCGCGTTTCC	Genotyping PCR of Δ*arr*$_{Mab}$
Mab_rpoB_F1	TTGTGATCGATCGGGTGTG	*rpoB* sequencing of RFB-R1
Mab_rpoB_R1	GACTTGTCGATGCTCTCGTCG	*rpoB* sequencing of RFB-R1
Mab_rpoB_F2	GTCGTGTCGCAGCTCGTC	*rpoB* sequencing of RFB-R1
Mab_rpoB_R2	ACGGTTACCGAAGTGGTCG	*rpoB* sequencing of RFB-R1
Mab_rpoB_F3	GCCAGACCACGATGACCG	*rpoB* sequencing of RFB-R1
Mab_rpoB_R3	CGAGACCTTCCGGTAAGG	*rpoB* sequencing of RFB-R1
Mab_rpoB_F4	CTCGCTGTCGGTGTACGC	*rpoB* sequencing of RFB-R1
Mab_rpoB_R4	GTCGGCGATGACCTGACC	*rpoB* sequencing of RFB-R1
Mab_rpoB_F5	AACCAGAAGCCCATCGTGG	*rpoB* sequencing of RFB-R1
Mab_rpoB_R5	TCACCGTCGGAGATCTTGC	*rpoB* sequencing of RFB-R1
Mab_rpoB_F6	CCGGCGTGAATGAGCTCG	*rpoB* sequencing of RFB-R1
Mab_rpoB_R6	ATCTCACCGAACCGCTGG	*rpoB* sequencing of RFB-R1

Mab_rpoB_F7	CGTACTCGATGATCACCCAGC	*rpoB* sequencing of RFB-R1
Mab_rpoB_R7	GGATGTCATCCGCCGACG	*rpoB* sequencing of RFB-R1

[a]Restriction sites are underlined
